# Development of a Rapid Sex Identification Method for Newborn Pigeons Using Recombinase Polymerase Amplification and a Lateral-Flow Dipstick on Farm

**DOI:** 10.3390/ani12212969

**Published:** 2022-10-28

**Authors:** Fang-Yu Lai, Kuang-Chih Chang, Chi-Sheng Chang, Pei-Hwa Wang

**Affiliations:** 1 Key Laboratory of Animal Genetics, Breeding and Bioresources, Department of Animal Science and Technology, College of Bioresources and Agriculture, National Taiwan University, Taipei City 10672, Taiwan; 2 Avance Technology Co., Ltd., 10F., No. 1, Ln. 83, Sec. 1, Guangfu Rd., Sanchong Dist., New Taipei City 24158, Taiwan; 3 Department of Animal Science, Chinese Culture University, No. 55, Hwa-Kang Rd., Yang-Ming-Shan, Taipei City 11114, Taiwan

**Keywords:** pigeons, sex identification, recombinase polymerase amplification-lateral-flow dipstick (RPA-LFD), on-farm test

## Abstract

**Simple Summary:**

The sex of a bird is important for aviculture, scientific research, and conservation. Sex identification is usually not easy, even if the bird’s appearances and sex organs are examined more closely. In monomorphic birds—or most birds during young, molecular sexing—there is a requirement for a fast and accurate identification method. We have designed a pair of DNA primers that is unique to the W chromosome of pigeon, which was unique to the female; further, RPA and LFD are combined for the purposes of a portable field detection for a sex identification method for birds (i.e., pigeons). The minimal-equipped on-farm approach was tested on pigeon sexing and the results have been 100% correct, so far. The concept of this study could spread to any kind of bird to meet the needs and achieve the goals of bird studies and businesses.

**Abstract:**

According to pigeon racing rules in Taiwan, the pigeon raiser must decide which juveniles will be chosen as soon as possible. Differentiating the sex of young pigeons based on appearances, and other traditional methods, can be time-consuming and require several pieces of equipment. Recombinase polymerase amplification (RPA) combined with a lateral-flow dipstick (LFD) could further simplify the presentation of amplification results. A designed reverse primer and probe were labeled with biotin and FAM (fluorescein), respectively, to serve as ligands in the LFD. With the addition of a designed forward primer, the RPA-LFD can be used to perform sex identification of pigeon DNA. The optimal conditions were determined to require at least 6.3 pg of the DNA template, a temperature of 37 °C, and a reaction time of at least 20 min. Under these conditions, the test band area on the strip appeared as a dark color if the sample contained female template DNA, whereas the male DNA samples did not produce any test signal in any of the conditions. The results of random samples using RPA-LFD under the optimal conditions agreed with the results of the same samples determined by PCR-agarose gel electrophoresis. The approach in this study represents a rapid and accurate method for pigeon sexing.

## 1. Introduction

The domestication of the domestic pigeon took place approximately 10,000 years ago, as a result, pigeons have played a role in the development of human history. In fact, pigeon keeping is a tradition that is older than Ancient Egypt. For thousands of years Egyptians have reared pigeons [1]. They are used for both sport and food [2]. Nowadays, due to the development of communication technology, the use of domestic pigeons for message transmission is rare. At present, a small number of pigeons are used for meat in Taiwan, but the main purpose of raising domestic pigeons is for use in competitions. In addition, flights of up to 1800 km have been reported in the United States [3].

Pigeon racing originated in Europe, specifically in Belgium, where it is also the most popular. The earliest appearance of pigeon racing in Taiwan can be traced back to the Japanese occupation period. After decades of evolution, pigeon racing in Taiwan has become increasingly complex. Pigeon racing in most countries is of a land-flying variety; further, each pigeon can participate in multiple races over a lifetime of 15–20 years, however this is not the case in Taiwan.

According to a documentary by the National Geographic Channel (National Geographic TW: Taiwan to the World: The Pigeon Game, (https://www.youtube.com/watch?v=vgb10o2fpX0, accessed on 7 August 2022), there are approximately 500,000 people involved in the pigeon industry in Taiwan; in addition, this demographic raises between 2 and 3 million pigeons annually. The pigeon racing system in Taiwan has two characteristics that make it one of the most unique systems in the world as: (a) Only young pigeons (3–4 month old) can participate in races, and a pigeon can only participate once in its life—regardless of whether it wins or loses. It makes it impossible for fanciers to accumulate a pigeon’s experience record for many years and many competitions, and it is, therefore, impossible to judge the skills and performance of the players through repeated practice. (b) The pigeons must be ringed within one week of birth in order to qualify as competitors. At this time, the owner can only judge whether the pigeon is suitable for competition based on the bloodline of the previous generation and his own experience. In the next phase, he must rely on breeding technology and training skills; further, he must report to the pigeon association after six months in order to confirm the qualification [4]. The experience and perception of pigeon farmers indicate that, in general, female pigeons are more likely to win in competitions than male pigeons. Therefore, pigeon farmers prefer to select female pigeons with an excellent bloodline at one week old for subsequent feeding and training.

Birds can be separated into two categories based on the appearances of male and female animals. Dimorphic birds, such as chickens, mandarin ducks, and peacocks etc., present obvious differences in the appearances of male and female animals. By contrast, among monomorphic birds, such as pigeons and parrots, the differences in the appearances between male and female animals can be difficult to identify, regardless of the maturity status of the bird. There are many methods to identify the sex of birds (see Table 1). If the male and female birds could be successfully identified, this would be beneficial for studies of animal populations, behavior, evolution, and wild animal management. Sex identification would also be beneficial for the analysis of breeding strategies, improve reproduction planning, and provide practical advantages for forensic cases. Consequently, various techniques have been developed for sexing pigeons, which have high economic value. Currently used traditional methods include (1) the identification of gonadal sex organs using laparoscopy or vent endoscopy; (2) phenotypic sexing based on slightly different morphological features; and (3) behavioral sexing, based on the different hormonally induced behaviors of male and female animals [5]. Moreover, vent sexing is an important method in newborn poultry. However, high accuracy is only achieved by highly trained technicians [6]. Immunoassay determination of the ratio of estrogen to testosterone in the blood or feces of sexually mature birds can also be used for sex determination. In general, female birds have a higher E/T ratio [7]. Due to the time-consuming and invasive nature of traditional methods, which also feature low accuracy, DNA sexing has, as a result, become a popular method. A comparison of monomorphic bird sexing methods based on limitations in use, condition, and application is shown in Table 1.

DNA-based sexing depends on genome-specific markers. The sex chromosome of birds contains a heterogametic sex chromosome (W and Z) in females and a homogametic sex chromosome (i.e., two Z) in males [8]. Sex differentiation can be performed based on variant genes, or DNA sequences when compared between the Z and W chromosomes. Chromodomain helicase DNA binding 1 (*CHD1*) was the first gene to be recognized as a sex-linked maker for the sex identification of non-ratite birds [9]. A conserved 0.6 kb *Eco*RI fragment (EE0.6) on the W chromosome has also been used for the sex determination of several bird species [10,11]. The PCR-based technique has been widely applied for performing sex differentiation among birds. The standard PCR approach involves the amplification of specific fragments using specific primers against the Z and W chromosomes, followed by an analysis of the fragments using electrophoresis [12]. Differences in the fragment numbers or lengths indicate differences in sex. In addition to the standard sexing method, several alternative PCR-based methods have been developed for specific conditions, including single-strand conformation polymorphism (SSCP), restriction fragment length polymorphism (RFLP), random amplified polymorphic DNA (RAPD), amplified fragment length polymorphism (AFLP), and microsatellite and high-resolution melting analysis (HRM) [13]. 

**Figure 1:**
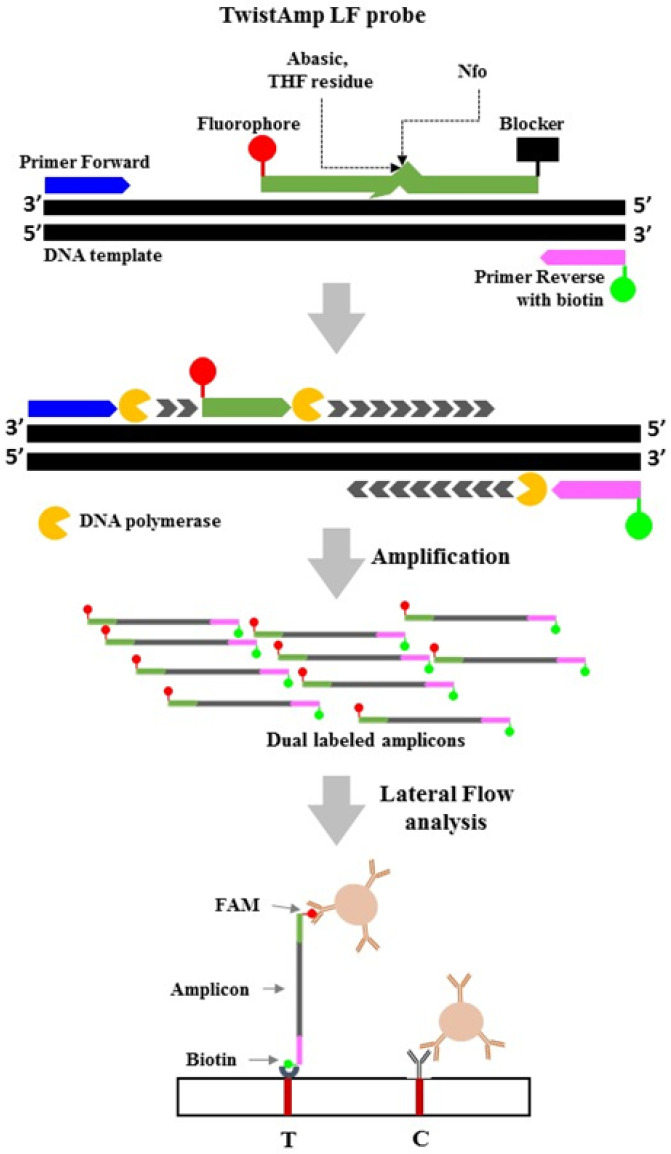
RPA-LFD reaction mechanism. The RPA followed a TwistAmp^®^ nfo kit. Amplification, with forward primer, labeled reverse primers and probe, occurred when endonuclease IV (nfo) recognizes and cleaves the tetrahydrofuran (THF) residue within the probe, detaching the 3′-end block. A double labeled amplicon was formed. The RPA amplicons travel in a buffer stream, which contains antibody-coated gold particles, to be trapped at the test line by biotin–ligands, resulting in an appearance of dark-purple color indicative of a positive result. Non-captured gold particles move through the test line to be fixed at the control line by anti-rabbit antibodies.

These PCR-based methods require complicated thermo-cycling processes and machines, in addition to the ability to perform agarose or capillary electrophoresis. To develop a fast and on-site sexing system, all electrical devices should be eliminated to the most maximum degree possible. Isothermal amplification methods are suitable for less well-equipped locations. Among existing isothermal amplification methods, loop-mediated isothermal amplification (LAMP) represents a popular method that was developed for the purposes of bird sexing [14,15,16]. The recombinase polymerase amplification (RPA), another isothermal technique, can also be used to amplify specific DNA within a single temperature range (37–42 °C) [17]. The mechanism underlying RPA is based on a natural cellular process known as homologous recombination. The reagents required to perform RPA include three proteins: recombinase, recombinase loading factor, and single-stranded binding protein. The recombinase T4 *Uvs*X, together with its cofactor *Uvs*Y, aggregates with two oligonucleotide primers in order to identify a homologous target sequence in a DNA template. Upon the identification of a specific homologous sequence, the complex forms a D-loop structure. One single strand of the D-loop is maintained as a single strand by the T4 gp32 protein. The other side of the D-loop undergoes strand displacement amplification by the *Bacillus subtilis* DNA polymerase I (*Bsu*) or *Staphylococcus aureus* polymerase (*Sau*) in order to generate a double-stranded DNA [18]. RPA products can be detected by agarose gel electrophoresis (AGE) [17] or in real-time, using TwistAmp^®^ exo probes [19]. RPA products can also be detected using a lateral-flow dipstick (LFD) assay [20]. The RPA-LFD assay starts with the primer pair, an oligonucleotide probe, and a DNA template. One of the primers and the probe are conjugated with biotin and a FAM (carboxyfluorescein) antigenic label, respectively. In addition, on the probe, a C3 spacer is attached to the 3′ end, and a base analog, tetrahydrofuran (THF), is inserted as an internal base. The resulting amplicons generated by RPA primers and the probe contain two labels on both sides and can be visualized using a “sandwich” assay [21]. The LFD assay only requires 5–10 min to be conducted and can be performed without access to any special equipment. When combined with RPA, the detection procedure can be further simplified. A diagram of the principle of the RPA-LFD assay is shown in Figure 1. In this study, we describe the development of a rapid and efficient method for the identification of a pigeon’s gender by combining RPA with LFD for amplification and detection, respectively.

RPA-LFD detection technology: (a) is real-time; (b) needs few molecular detection instruments (e.g., PCR or electrophoresis equipment); and (c) can be used for on-farm detection. In Taiwan, if pigeons are to be qualified as racing pigeons in the future, the pigeon farmer needs to determine the sex of the male and female by their appearance within one week of age. This is due to the fact that pigeons must be ringed in the first week of life, so that selecting the females with a good bloodline for further feeding and training can be conducted. Therefore, it is extremely important to develop a low-cost real-time method that can determine the sex on the farm without the need for many analytical instruments; further, it must come with highly accurate results. To the best of our research team’s knowledge, the use of RPA-LFD is a method that can meet the above requirements. Moreover, this study aims to test whether RPA-LFD can perform functional gene testing on a farm.

## 2. Materials and Methods

### 2.1. Animals and Sample Collection

Blood or feather samples were collected from 3 pigeon flocks maintained on various private farms (i.e., 3 farms) located in different regions of Taiwan. For blood collection, 0.2–0.3 mL of blood was drawn from the superficial plantar metatarsal vein or wing vein using a 1 mL syringe and 23 G needles (TERUMO^®^, TERUMO Co., Laguna, Philippines), and transferred to a blood collection tube containing the anticoagulant EDTA K3 (BD, Becton, Dickinson and Company, Franklin Lakes, NJ, USA). For feather collection, a 0.5–1-cm of the calamus section was cut starting from the inferior umbilicus and placed in a 1.5 mL eppendorf tube. All animal experiments were approved by the Institutional Animal Care and Use Committee of Avance Technology Co., Ltd. (Protocol number: AT Ltd. No. 2018-001).

### 2.2. Total DNA Template Preparation

Whole-genomic DNA from EDTA-anticoagulated blood was extracted using a Genomic DNA Isolation Reagent (GenePure Technology CO., LTD, Taichung, Taiwan). Further, 10~15 μL of blood was mixed with 1 mL of the reagent. For higher purity, the mixed solution was completely mixed using phenol–chloroform and then precipitated with isopropanol. Total DNA from the feather samples was extracted using a QIAamp DNA Mini Kit (Qiagen, Courtaboeuf, France), following the manufacturer’s instructions. Two to five feather fragments were soaked in the kit’s reagent with proteinase K at 56 °C for 1 h to overnight; then, the liquid was passed through the column by centrifugation. The concentration and quality of DNA were determined using a NanoDrop 1000 Spectrophotometer (Thermo Fisher Scientific Inc., Waltham, MA, USA), at 260 and 280 nm, and then adjusted to a concentration of 50 ng/μL using DNase-free water. The quantified DNA was stored at −20 or −80 °C refrigerator until RPA-LFD was performed.

### 2.3. Recombinase Polymerase Amplification Primer and Probe Design

According to the Appendix of the TwistAmp^®^ reaction kit manual http://www.twistdx.co.uk/wp-content/uploads/2021/04/twistamp-assay-design-manual-v2-5.pdf, accessed on 7 August 2022), a series of suitable (29–35 bp, GC content 30–70%) candidate RPA primers were designed using Primer3 (https://www.primer3plus.com, accessed on 7 August 2022) [22] in order to target the conserved regions of the *CHD* gene sequences found on the Z (GenBank accession number: GU289184.1) and W (GenBank accession number: GU289183.1) chromosomes. The forward primer, P01 (5′-CTCCCAAGGATGAGGAACTGTGCAAAACAGG-3′), and the reverse primer, P02 (5′-biotin- GATATGGAGTCACTATCAGATCCAGAGTATC-3′), were both designed to target a conserved region of *CHD* Z and *CHD* W. A probe for the LFD assay was added to the reaction of the TwistAmp^®^ nfo kit (TwistDx, Cambridge, UK), with two primers. The LFD probe was designed based on the gene sequence between the designed RPA primers. The probes consisted of an upstream stretch (30-nt) carrying a 5′-FAM antigenic label that was connected using a THF spacer to an adjacent downstream oligonucleotide (15-nt) carrying a C3-spacer (polymerase extension blocking group) at the 3′ end. The probe, referred to as Wprobe (5′-FAM-ATAGCACATTATTAAAATGTTTTAGTCACA-THF-AGCTTTGAACTACTT-C3-spacer-3′), used in this study only targeted the *CHD* W sequence between P01 and P02. All primers and the probe were synthesized by Integrated DNA Technologies (Coralville, IA, USA).

### 2.4. Recombinase Polymerase Amplification

To test the suitability of the primers, RPA was performed in a 50 μL volume using a TwistAmp^®^ Basic kit (TwistDx, Cambridge, UK). The mixtures contained 10–50 ng of template DNA, 0.2 μM of each RPA primer, 1× rehydration buffer, and DNase-free water. A dry enzyme pellet was then added and thoroughly mixed. A total of 14 mM magnesium acetate was pipetted into the tube lids, followed by centrifugation to transfer the magnesium acetate into the tube in order to initiate the RPA mechanism at 40 °C for 20 min. The reactions were visualized by 2% agarose gel electrophoresis (AGE). For the purposes of probe screening, RPA was performed in a 50 μL volume using a TwistAmp^®^ nfo kit (TwistDx, Cambridge, UK). The P01 and P02 primers were adapted for the RPA assay by labeling the reverse P02 primer with a 5′ biotin residue and testing both primers for compatibility with internal RPA LF (lateral-flow) probes, which were designed according to the TwistDX guidelines. A solution containing 0.2 μM P2 primer, 0.1 μM P1 primer, and 0.12 μM LF probe in 1× rehydration buffer and DNase-free water was added to a dry pellet from the Twist Amp nfo kit (TwistDx, Cambridge, UK). The template DNA (1 ng) was added, and 14 mM of magnesium acetate was pipetted into the tube lids. Centrifugation was performed to initiate the RPA mechanism at 40 °C for 20 min. The reactions were then visualized by LFD.

### 2.5. Lateral-Flow Dipstick (LFD) Assay

To detect the RPA amplicon by LFD (Milenia Biotec, Giessen, Germany), 1 μL of the RPA product was transferred to a new tube containing 100 μL of the assay buffer (1× phosphate-buffered saline with 0.1% Tween 20). The LFD strip was then dipped into the mixture for 5 min to visualize the test result. The entire LFD assay was performed at room temperature.

### 2.6. Evaluation of the Optimal Conditions of RPA-LFD

The RPA in molecular sexing was first performed at 34, 37, 40, and 43 °C in order to determine the optimal reaction temperature. As the temperature was determined, the optimal reaction time for the RPA was performed for 5, 10, 15, 20, 25, 30, 40, and 60 min at the proper temperature. After the conditions of RPA were set, four-fold serial dilutions of the total DNA extracted from both a female pigeon and a male pigeon, at 100 ng, 25 ng, 6.3 ng, 1.6 ng, 0.4 ng, 0.1 ng, 0.025 ng, 6.3 pg, 1.6 pg, and 0.4 pg, were used as templates for the RPA reaction. The DNA products from the RPA were then analyzed by LFD.

### 2.7. Reliability and Positive Rate Comparison between RPA-LFD and PCR-AGE

The sex testing of samples from pigeons with unknown genders was conducted using both the RPA-LFD and AGE assays, as follows: (1) The RPA-LFD test was performed according to the previously described procedures for the sex identification of field samples using genomic DNA. (2) PCR was performed on a 20 µL volume using a thermal cycler (GeneAmp PCR system 9700, Applied Biosystems, Foster City, CA, USA) containing 0.5 U *Taq* DNA polymerase (TAKARA, Kusatsu, Japan); 1× PCR buffer (1.5 mM MgCl_2_); 0.2 mM dNTP, 0.2 µM forward and reverse primers [23]; and 50 ng gDNA. The PCR cycling program was as follows: 95 °C for 5 min, 35 cycles of 95 °C for 30 sec, 50 °C to 65 °C for 40 sec, 72 °C for 50 sec, and a final elongation step at 72 °C for 7 min. The amplified PCR products were analyzed with AGE, as previously described.

### 2.8. Statistical Analysis

The differences between the two detection methods were judged with Cohen’s kappa coefficient [24]. The analytical value κ value ranges from 0–1. The value closer to 1 indicates higher consistency.

## 3. Results

### 3.1. Determination of Optimal RPA-LFD Conditions

**Figure 2:**
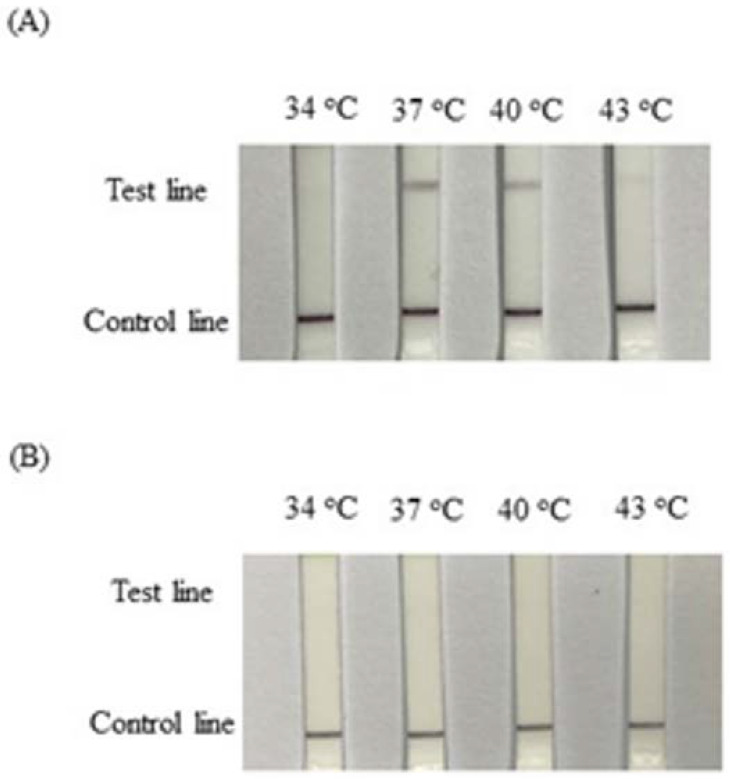
Optimization of the RPA-LFD reaction performed at different temperatures. (**A**) Reactions containing 0.1 ng/μL of genomic DNA from a female pigeon performed at different temperatures for 20 min. (**B**) Reactions containing 0.1 ng/μL of genomic DNA from a male pigeon performed at different temperatures for 20 min.

**Figure 3:**
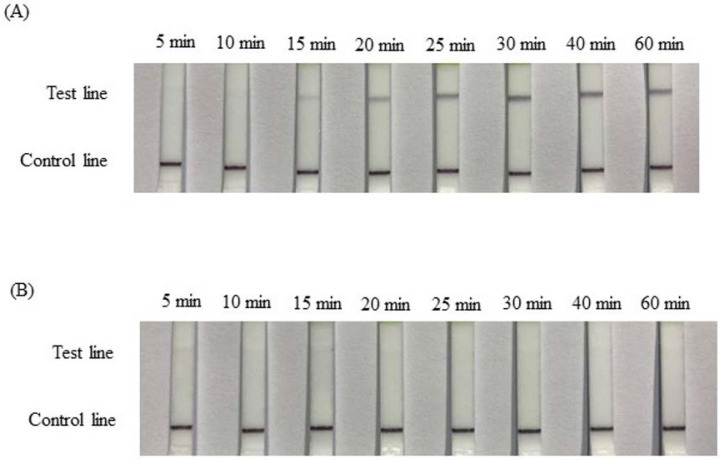
Optimization of RPA-LFD at different reaction times. (**A**) Reactions containing 0.1 ng/μL of genomic DNA from a female pigeon performed at 37 °C for different reaction times. (**B**) Reactions containing 0.1 ng/μL of genomic DNA from a male pigeon performed at 37 °C for different reaction times.

The proper temperature for the RPA reactions was determined using 0.1 ng of genomic pigeon DNA as a template. The amplification was performed for 20 min at 34, 37, 40, and 43 °C to determine the optimal reaction temperature (Figure 2). The darkest signal appeared on strips following reactions performed at 37 °C using the female sample (Figure 2A). The depth color for the band that formed after the 40 °C reaction was slightly lighter than that formed after the 37 °C reaction. No signals were detected on strips used to test the male samples at any temperature (Figure 2B). Based on these results and the manufacturer’s instructions, 37 °C could be selected as the assay temperature. To determine the optimal reaction time for the RPA-LFD test, the RPA reaction was performed for 5, 10, 15, 20, 25, 30, 40, and 60 min at 37 °C (Figure 3A). In the female samples, the quantities of the amplification products detected after reaction times of 25, 30, 40, and 60 min were similar but higher than those obtained after reaction times of 15 and 20 min. The amount of amplification product detected on the LF strips gradually decreased from 25 min to 15 min and totally disappeared when the reaction time was shorter than 15 min. An incubation time of 20–30 min was selected as optimal. In male samples, no signals were detected on any LFD strips for the reaction times ranging from 5 min to 60 min (Figure 3B).

### 3.2. The Sensitivity of the RPA-LFD Assay

**Figure 4:**
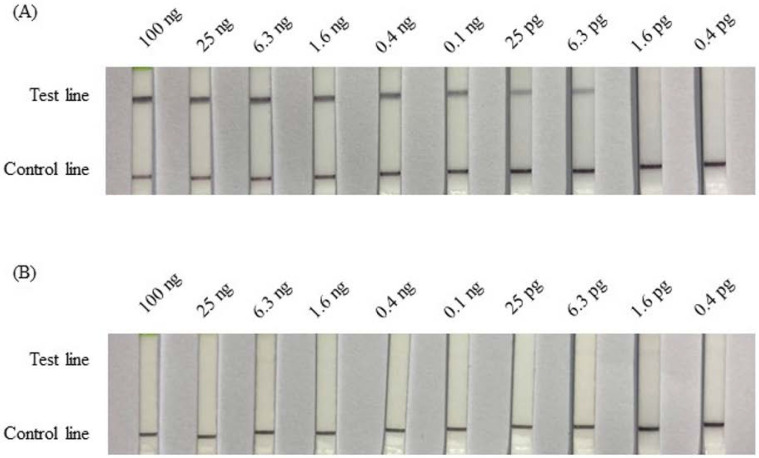
Molecular sensitivity test results for the RPA-LFD assay using 4-fold serially diluted total DNA extracted from pigeons as the template. (**A**) DNA template from a female pigeon. (**B**) DNA template from a male pigeon. In addition, a 100 ng, 25 ng, 6.3 ng, 1.6 ng, 0.4 ng, 0.1 ng, 25 pg, 6.3 pg, 1.6 pg, and 0.4 pg dilution series of total DNA was prepared from female and male pigeons.

The sensitivity of the RPA-LFD assay was tested using 4-fold serial dilutions of total DNA at concentrations ranging from 100 ng to 0.4 pg, and was performed at 37 °C for 25 min. In the female samples (Figure 4A), the test lines appeared from 100 ng to 6.3 pg. No bands appeared when the total DNA was less than 1.6 pg. A detection limit of 6.3 pg total DNA was identified for female pigeon samples. In male samples (Figure 4B), no visible bands were detected for any amount of total DNA, ranging from 100 ng to 0.4 pg.

### 3.3. Confirmation of the RPA-LFD Assay Reliability Using PCR-AGE

**Figure 5:**
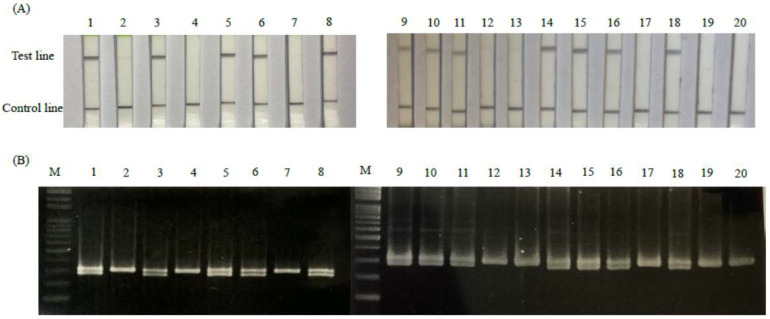
Molecular reliability of the RPA-LFD method for pigeon sex identification. (**A**) The results are visualized by LFD. (**B**) The results for the same samples were visualized by PCR-AGE. Lanes M: 100 bp ladder molecular marker. In addition, 1–20 were 20 random samples selected from samples of 20 pigeons (from different lots).

To confirm the validity and reliability of the RPA-LFD assay, RPA-LFD was performed in parallel with the traditional PCR-AGE assay. A total of 20 random samples, comprising 12 females and 8 males, were assessed by both RPA-LFD and PCR-AGE assays (Figure 5A). Samples 1, 3, 5, 6, 8, 9, 10, 11, 14, 15, 16, and 18 were detected as female pigeons by the RPA-LFD assay, which were the same results obtained using PCR-AGE (Figure 5B). In the PCR-AGE results, the female samples produced two bands near the 300 bp molecular marker, whereas the male samples only produced one band. Two bands were detected in samples 1, 3, 5, 6, 8, 9, 10, 11, 14, 15, 16, and 18 in the PCR-AGE assay, which were the same samples identified as female by RPA-LFD. The remaining eight samples of Figure 5 were newborn pigeons, and the others were adults. All results were analyzed via Cohen’s kappa coefficient test, and the test result (κ = 1) showed that both detection results were almost the same (κ > 0.95).

## 4. Discussion

Methods for the isothermal amplification of nucleic acids have been developed since the early 1990s. These approaches represent simpler and more efficient tools than typical PCR methods for molecular biology applications. To date, more than ten methods of isothermal amplification have been developed, some of which have been successfully commercialized [25]. The LAMP method has been the most popular method through 2018 (based on data from an isothermal nucleic acid amplification technology market analysis, according to the technological method [NASBA, HDA, LAMP, SDA, SPIA, NEAR, TMA, RCA, RPA, and SMAP], and segment forecasts it will also be the most popular for 2020–2027, https://www.datamintelligence.com/research-report/isothermal-nucleic-acid-amplification-technology-market, accessed on 7 August 2022). However, RPA features several advantages when compared with both conventional PCR-based methods and LAMP assays [17]. First, this technique amplifies DNA at constant and relatively low temperatures (optimal range: 37–42 °C), which eliminates the need for thermal cyclers. Second, each RPA assay requires only one pair of oligonucleotide primers, whereas the LAMP technique requires four to six primers to synthesize various sizes of DNA amplicons [26]. Third, RPA amplicons can be visualized in real-time using LFDs.

The reaction conditions for RPA suggested in the official manual were 2.1 µL of each forward and reverse primer (10 µM). The probe (10 µM) volume was suggested at 0.6 µL, resulting in a volume ratio of 7:7:2. In our tests, the optimal ratio of the forward primer, reverse primer, and probe was 5:10:6 (data not shown). In general, initial experimental studies show that the reaction temperature in the manual was recommended at 37–39 °C; further, some articles have described the reactions being performed at 37–42 °C. In the present study, 34, 37, 40, and 43 °C were attempted. Almost no signal could be detected in reactions performed at 34 and 43 °C, and the darkest band was identified for the reaction performed at 37 °C. Li et al. [27] attempted reaction temperatures of 30, 35, 37, 39, 45, and 50 °C for the detection of *Salmonella* in food using RPA-LFD. Similar to our results, the strongest test lines were obtained at 35, 37, and 39 °C. When the temperature exceeded 39 °C, the test bands gradually became weaker. A similar result was observed for the caprine arthritis and encephalitis virus (CAEV) tested using RPA-LFD [28]. In another study, RPA-LFD was applied for the detection of the *Pasteurella multocida* pathogen in cattle. The test band was brightest between 39 and 42 °C [29]. Although the results of optimal temperature tests have varied across detection targets, the optimal outcomes have always been reported between 37 and 42 °C, which may be associated with the primer design. Thus, in our study, 37 °C was determined to be the optimal assay temperature. The reaction time was examined at 37 °C, ranging from 5 to 60 min. In female samples, the test band appeared after a reaction time of approximately 15 min and became darkest after 25 min (Figure 2A). In a study examining the detection of *Mycoplasma ovipneumoniae* infections using RPA-LFD, the darkest band was obtained at 39 °C using 0.4 mM of both probes and 0.12 mM probe after a 10 min reaction, which was earlier than the present test (for which a 15 min reaction was necessary [30]). For the detection of *Salmonella,* the test band appeared after 5 min and became the darkest after 20 min at 39 °C, with 0.4 µM of both primers and 0.1 μM probe [27]. Our delayed reaction time (15 min) may be due to the use of an insufficient primer quantity (0.1 μM and 0.2 μM). After a 25 min reaction time, however, the female signal was clearly visible. This may be due to the slightly higher probe quantity used (0.12 μM) in the reaction. If the reaction time requires shortening, this may be possible by increasing the primer quantity. The sensitivity of RPA-LFD for sex identification was tested using different concentrations of DNA templates from female and male pigeons, ranging from 100 ng/μL to 0.4 pg/μL, using intervals obtained by performing 4-fold serial dilutions. The test bands in the female samples gradually lightened with decreasing amounts of the DNA template. The signals were visible until less than 6.3 pg was used (Figure 3A). In the male samples, no signal was detected for any quantity, even on the 100 ng strip (Figure 3B). The sensitivity of the RPA-LFD for different DNA resources has varied, reported at values of 0.2 ng, 80 pg, 10 fg and 50 pg [28,30,31,32]. The RPA-LFD detector used in this study, displayed a higher sensitivity than many other similar techniques. Primers P01 and P02 were 31 bp each and can produce an approximately 265 bp band from the W chromosome or a 280 bp band from the Z chromosome, based on the PCR results performed using total DNA from pigeons [23]. The 20 random pigeon DNA samples were amplified by PCR using the primers P8 and CHR-R, and the products were separated by AGE. The results indicated 12 females and 8 males, which was confirmed by the pigeon’s owners. The test band identified by the RPA-LFD matched the result of the PCR-AGE, with samples 1, 3, 5, 6, 8, 9, 10, 11, 14, 15, 16, and 18 revealing clear bands, indicating female samples, whereas samples 2, 4, 7, 12, 13, 17, 19, and 20 showed no signal, indicating male samples (Figure 4). The accuracy of the RPA-LFD for sex identification was 100%. The determination of pigeon sex by RPA-LFD was demonstrated to be a high-accuracy, fast (real-time), on-farm test that uses few analytical instruments. As such, it is an easy sexing method.

Whether the extraction of genomic DNA from blood or feathers depended on farmers’ demand. One drop of blood, taken by lancet stabbing on pigeon’s toes, can easily extract a greater amount of DNA than can be obtained from feathers; further, the invasion to the animal is minimal. In addition, the extraction of DNA from larger feathers, of which the calamus is full of tissue and blood, is an easier way for the purposes of extraction in regard to young pigeons.

## 5. Conclusions

Correctly identifying the sexes of young pigeons is necessary to comply with racing rules in Taiwan. The traditional approaches used for sex identification require complex procedures and can be time-consuming when using molecular biology techniques and instruments in the laboratory. In this study, proper primer and probe design, as well as optimal reaction conditions, made the RPA-LFD an efficient method for performing sex identification in pigeons in the fields and in farms. In the heavily competitive racing environment, racing pigeon owners can use the RPA-LFD kit to easily, rapidly, and accurately identify the sexes of newborn pigeons (before they are 1 week of age), which can benefit pigeon owners in the training and breeding of pigeons. Many reports show that the RPA-LFD detection technology is widely used for rapid point-of-care detection of pathogenic bacteria in humans and animals. This research report is the first report that uses RPA-LFD to perform rapid on-farm detection of functional genes (e.g., sex chromosome gene-*CHD* gene), instead of rapid point-of-care detection of pathogens in livestock, poultry, and humans.

## Figures and Tables

**Table 1:** Comparison of sexing methods used for monomorphic birds.

Methods	Limitation of Age	Limitation of Breeding Periods	Training of Technicians	Invasion	Stress of Birds	Onsite
Morphology	Yes	No	Less	No	With the photographic method can be exempted	Yes
Behavior	Yes	Yes	Yes	No	No	Yes
hormone analysis	Yes	No	Less	Less	Yes	No
Laparoscopy	Yes	Yes	Yes	Yes	Yes	No
vent test	Yes	Yes	Yes	Less	Yes	Yes
Molecular marker	No	No	Less	Less	Yes	No

## Data Availability

All relevant data are included in this paper. The datasets generated during and/or analyzed during the current study are available from the corresponding author upon request.

## References

[1] Blechman A.D. (2007). Pigeons: The Fascinating Saga of the World’s Most Revered and Reviled Bird.

[2] Gilbert M.T.P., Shapiro M.D., Smith C. (2014). Pigeons: Domestication. Encyclopedia of Global Archaeology.

[3] Walcott C. (1996). Pigeon homing: Observations, experiments and confusions. J. Exp. Biol..

[4] (2009). Taiwan to the World: The Pigeon Game. National Geographic Society. https://www.youtube.com/watch?v=vgb10o2fpX0.

[5] Miąsko M., Gruszczyńska J., Florczuk P., Matuszewski A. (2017). Determining sex in pigeons (*Columba livia*). World Sci. News..

[6] Cerit H., Avanus K. (2007). Sex identification in avian species using DNA typing methods. World’s Poult. Sci. J..

[7] Bercovitz A.B., Czekala N.M., Lastey B.L. (1978). A new method of sex determination in monomorphic birds. J. Zoo Anim. Med..

[8] Ezaz T., Stiglec R., Veyrunes F., Marshall Graves J.A. (2006). Relationships between vertebrate ZW and XY sex chromosome systems. Curr. Biol..

[9] Ellegren H. (1996). First gene on the avian W chromosome (CHD) provides a tag for universal sexing of non-ratite birds. Proc. R. Soc. Lond B Biol. Sci..

[10] Ogawa A., Solovei I., Hutchison N., Saitoh Y., Ikeda J.E., Macgregor H., Mizuno S. (1997). Molecular characterization and cytological mapping of a non-repetitive DNA sequence region from the W chromosome of chicken and its use as a universal probe for sexing *Carinatae* birds. Chromosom. Res..

[11] Lin E.C., Hsu H.A., Chao M.C., Chan F.T., Wang L.M., Tsao H.S., Chang C.H., Lin P.Y., Wang B.J., Yuan H.W. (2011). Application of *CHD1* gene and EE0.6 sequences to identify sexes of several protected bird species in Taiwan. Taiwania.

[12] Liang S.J., Chen M.X., Gao C.Q., Yan H.C., Zhang G.L., Wang X.Q. (2019). Sex identification of pigeons using polymerase chain reaction analysis with simple DNA extraction. Avian Biol. Res..

[13] Morinha F., Cabral J.A., Bastos E. (2012). Molecular sexing of birds: A comparative review of polymerase chain reaction (PCR)-based methods. Theriogenology.

[14] Chan K.W., Liu P.C., Yang W.C., Kuo J., Chang C.L.T., Wang C.Y. (2012). A novel loop-mediated isothermal amplification approach for sex identification of *Columbidae* birds. Theriogenology.

[15] Centeno-Cuadros A., Tella J.L., Delibes M., Edelaar P., Carrete M. (2017). Validation of loop-mediated isothermal amplification for fast and portable sex determination across the phylogeny of birds. Mol. Ecol. Resour..

[16] Koch H.R., Blohm-Sievers E., Liedvogel M. (2019). Rapid sex determination of a wild passerine species using loop-mediated isothermal amplification (LAMP). Ecol. Evol..

[17] Piepenburg O., Williams C.H., Stemple D.L., Armes N.A. (2006). DNA detection using recombination proteins. PLoS Biol..

[18] Li J., Macdonald J., von Stetten F. (2018). Review: A comprehensive summary of a decade development of the recombinase polymerase amplification. Analyst.

[19] Euler M., Wang Y., Otto P., Tomaso H., Escudero R., Anda P., Hufert F.T., Weidmann M. (2012). Recombinase polymerase amplification assay for rapid detection of *Francisella tularensis*. J. Clin. Microbiol..

[20] Rosser A., Rollinson D., Forrest M., Webster B.L. (2015). Isothermal recombinase polymerase amplification (RPA) of Schistosoma haematobium DNA and oligochromatographic lateral flow detection. Parasites Vectors.

[21] Ghosh D.K., Kokane S.B., Kokane A.D., Warghane A.J., Motghare M.R., Bhose S., Sharma A.K., Reddy M.K. (2018). Development of a recombinase polymerase based isothermal amplification combined with lateral flow assay (HLB-RPA-LFA) for rapid detection of “*Candidatus Liberibacter asiaticus*”. PLoS ONE.

[22] Untergasser A., Nijveen H., Rao X., Bisseling T., Geurts R., Leunissen J.A. (2007). Primer3 Plus, an enhanced web interface to Primer3. Nucleic Acids Res..

[23] Lee J.C.I., Tsai L.C., Kuan Y.Y., Chien W.H., Chang K.T., Wu C.H., Linacre A., Hsieh H.M. (2007). Racing pigeon identification using STR and chromo-helicase DNA binding gene markers. Electrophoresis.

[24] Cohen J. (1960). A Coefficient of Agreement for Nominal Scales. Educ. Psychol. Meas..

[25] Zhao Y., Chen F., Li Q., Wang L., Fan C. (2015). Isothermal amplification of nucleic acids. Chem. Rev..

[26] Notomi T., Okayama H., Masubuchi H., Yonekawa T., Watanabe K., Amino N., Hase T. (2000). Loop-mediated isothermal amplification of DNA. Nucleic Acids Res..

[27] Li J., Ma B., Fang J., Zhi A., Chen E., Xu Y., Yu X., Sun C., Zhang M. (2020). Recombinase polymerase amplification (RPA) combined with lateral flow immunoassay for rapid detection of *Salmonella* in food. Foods.

[28] Tu P.A., Shiu J.S., Lee S.H., Pang V.F., Wang D.C., Wang P.H. (2017). Development of a recombinase polymerase amplification lateral flow dipstick (RPA-LFD) for the field diagnosis of caprine arthritis-encephalitis virus (CAEV) infection. J. Virol. Methods.

[29] Zhao G., He H., Wang H. (2019). Use of a recombinase polymerase amplification commercial kit for rapid visual detection of *Pasteurella multocida*. BMC Vet. Res..

[30] Gupta S.K., Deng Q., Gupta T.B., Maclean P., Jores J., Heiser A., Wedlock D.N. (2021). Recombinase polymerase amplification assay combined with a dipstick-readout for rapid detection of *Mycoplasma ovipneumoniae* infections. PLoS ONE.

[31] Dai T., Hu T., Yang X., Shen D., Jiao B., Tian W., Xu Y. (2019). A recombinase polymerase amplification-lateral flow dipstick assay for rapid detection of the quarantine citrus pathogen in China, *Phytophthora hibernalis*. PeerJ.

[32] Nie Z., Lü P., Zhang R., Tu Y., Liu Z., Li Y., Tang C., Li X., Zhao K., Zhou Q. (2021). A simple and rapid method for fish sex identification based on recombinase-aided amplification and its use in *Cynoglossus semilaevis*. Sci. Rep..

